# From Models to Implants: The Expanding Role of 3D Printing in Orthopedic Care

**DOI:** 10.7759/cureus.97992

**Published:** 2025-11-28

**Authors:** Ibrahim K Al Abid, Wasim I Alghoul, Ayman A Agha, Radwan A Aloti, Malak M Abedi, Mohamed T Abdelfattah, Ahmad Kharoufeh, Ahmad Omari, Mohamedanas Mohamedfaruk Patni

**Affiliations:** 1 Orthopedics, RAK Medical and Health Sciences University, Ras Al Khaimah, ARE; 2 Community Medicine, RAK Medical and Health Sciences University, Ras Al Khaimah, ARE

**Keywords:** 3d printing in orthopedics, electron beam melting, orthopedic procedures, orthopedic surgical repair, selective laser melting, surgical orthopedics

## Abstract

For decades, orthopedic implants have been essential for improving mobility, functionality, soothing pain, and rebuilding complex skeletal structures. Old manufacturing methods, such as casting, forging, and machining, have resulted in long-lasting implants but could not adapt to the complex anatomical variability in patients' anatomy. Additive manufacturing (3D printing) overcomes this limitation by allowing for complex, patient-specific computer-aided design (CAD) models to be created and implants to be manufactured containing precise porous architectures that encourage osseointegration and vascularization. This narrative review compiles evidence from systematic reviews, clinical studies, and experimental trials that were published between January 2020 and October 2025 to describe the historical development, clinical applications, outcomes, challenges, and future aspects of 3D-printed orthopedic implants. 3D printing is changing from an experimental modeling method to standard clinical practice with application in arthroplasty, spine surgery, trauma fixation, and oncology. The most common fabrication techniques for metallic implants can be divided into two components: selective laser melting and electron beam melting, while stereolithography and fused deposition modeling serve as significant aspects for the preparation of anatomical models, surgical guides, and biodegradable implants. Titanium alloys continue to be recognized as the gold standard for load-bearing devices, while biodegradable polymers and composites are being used in more recent pediatric and temporary implant surgeries. All data reports reduced operative time, better alignment precision, quicker osseointegration, and stable fixation with satisfactory short- to mid-term follow-up outcomes. Evidence from case series and systematic reviews supports the application of 3D printing in revision hip arthroplasty, cervical cages, patient-specific plates, and tumor reconstruction. However, cost, manufacturing standardization, reproducibility, infection control, and lack of substantial randomized clinical trials to prove the long-term safety and reliability of this new field still remain an issue that needs to be resolved to allow centers around the globe to adapt these kinds of techniques. Finally, these 3D-printed orthopedic implants present advanced anatomical integrity with greater mechanical stability than traditional types of implants. Moreover, innovative advances in technology include AI-assisted design, shape-morphing devices, and bioprinting of vascularized bone structures. Broader clinical adoption will depend on larger trials demonstrating safety, reproducibility, and cost-effectiveness.

## Introduction and background

Orthopedic implants have long been crucial for restoring functional mobility and relieving pain in patients with musculoskeletal disorders. However, conventional manufacturing methods such as casting and forging produce standardized implants that rarely match individual anatomy, contributing to complications such as loosening, infection, and decreased long-term Osseo-integration, which is the process of bone attaching directly to the implant surface. Worldwide, over two million orthopedic implant surgeries are performed annually, with revision rates of 5%-10% due to loosening, infection, or poor anatomical fit. These complications increase healthcare costs and patient disability, emphasizing the need for improved, patient-specific implant solutions. The emergence of additive manufacturing (3D printing), which is defined as the creation of parts layer by layer from design data, offers a transformative solution enabling personalized, patient-specific implants with improved integration and function [1]. Additive manufacturing is a new paradigm in medicine. Unlike subtractive modalities, which remove material from a bulk block, 3D printing constructs implants layer by layer within the framework of computer-aided design (CAD) models, which is a software that creates 3D implant models from patient scans (e.g., CT or MRI) [2]. It also allows the development of implants with intricately structured internal geometries, such as trabecular, porous structures reflecting cancellous bone, improving superior bone integration with implants [3]. The clinical justification of 3D printing in orthopedics is extensive, such as customization, because it provides a close fit to the patient’s body size. Improved osseointegration is achieved because it consists of porous scaffolds, which are 3D-printed lattice structures that allow bone and blood vessel growth, encouraging vascular and bone formation. It also requires less time to perform a surgical operation and allows rapid prototyping by enabling surgeons to practice difficult procedures with anatomic models. Finally, it is cost-efficient for complicated designs that would be unprofitable using normal manufacturers [4].

Additive manufacturing is no longer limited to an experimental setting. It is now used in everyday arthroplasty, spinal work, trauma fixations, tumor reconstruction, and the pediatric orthopedic field and has been reported to show early and favorable clinical effects [5,6]. But the field is currently evolving, and it is apparent that we still need higher level evidence on a broad clinical scale to consolidate current data, identify gaps in high-level clinical evidence, and highlight directions for standardized adoption and large-scale evaluation of 3D-printed orthopedic implants. There have been several important stages to the evolution of three-dimensional (3D) printing in orthopedics. Application in practice in the early part of the twenty-first century originally was limited to the creation of anatomical models and preoperative guides, constrained by material strength and biocompatibility [7]. Advanced imaging segmentation methods and CAD software were then employed to more accurately replicate the patient anatomy, a valuable step in the direction of clinical applications [8]. Initially, these issues, such as the poor mechanical performance of polymer-based models and the absence of medical-grade metals, were reduced by the introduction of selective laser melting (SLM) and electron beam melting (EBM) techniques, permitting the construction of durable titanium alloy implants [9]. This has developed 3D printing from a “lab” tool into a novel approach to clinical technology that can support patient-specific implant development, surgical rehearsal, and complex reconstruction across multiple orthopedic subspecialties. In this review, we provide an overview of existing knowledge on 3D-printed orthopedic implants, including their process from their genesis to the evolution of technology and material, applications to the clinic, their clinical outcome, problems, and future directions. The review aims to (1) provide a chronological introduction of implant fabrication, (2) contrast the clinical characteristics of 3D-printed versus conventional implants, (3) identify current reproducibility, regulatory, and cost issues, and (4) draw attention to ongoing trends such as artificial intelligence (AI) integration as well as bioprinting.

Methodology

A structured narrative review was performed using PubMed, Scopus, and Google Scholar. The search covered publications between January 2020 and October 2025. Search terms included combinations of "3D printing in orthopedics," "additive manufacturing," "orthopedic implants," "osseointegration," "bioprinting," and "patient-specific implants." Boolean operators ("AND," "OR") were applied to refine searches, and reference lists of relevant articles were manually screened for additional studies. Only peer-reviewed systematic reviews, clinical trials, case series, and experimental studies on orthopedic implant design, application, or outcome were included. Non-English publications and papers without clinical significance were excluded. Data were collated qualitatively, reflecting study heterogeneity, with emphasis on comparisons between outcomes, material properties, and emerging technologies. No formal risk‐of‐bias tool was used, but a greater focus was directed to more high‐level evidence-based systems, such as systematic reviews and meta‐analyses, where available.

## Review

Technological evolution of 3D printing in orthopedics

The development of orthopedic implant manufacturing has been a long-term improvement toward a solution of previous issues, from the fixed nature and little customization of original casting and forging processes and computer software to the precision of CAD/CAM, and finally to additive manufacturing (AM). The different stages dealt with different shortcomings: CAD/CAM enhanced reproducibility but failed to enable internal porosity for osseointegration, and AM was responsible for developing complex lattice designs and patient-specific geometries that ultimately paved the way for improved implant integration and stability.

Early Methods

Implant design used to be cast in casting/forging systems first, followed by milling to provide machined implants with high tolerances. These were all good, but the processes were limited by their inefficiencies: implant designs were standard, and modifying the design for specific individuals was out of the question.

The Digital Transition

CAD and CAM systems (late twentieth century) were developed, enhancing implant reproducibility and standardization, yet still adhered to a subtractive model where complex porous architectures that are key to bone integration could never be reached [7].

Emergence of AM

Rise of AM 3D printing was first introduced in orthopedics in the twenty-first century, initially as a surgical rehearsal for anatomical modeling. These advances in medical imaging and segmentation techniques in medicine enabled the accurate and 3D reconstruction of patient anatomy [8]. Early clinical applications were with regard to surgical guides for osteotomies and trauma fixation. As applications advanced in the clinic, these practices became definitive implants, which were especially useful for revision hip arthroplasty, complex fractures, and spinal reconstruction.

Adoption of Clinical Practice

**Figure 1:**
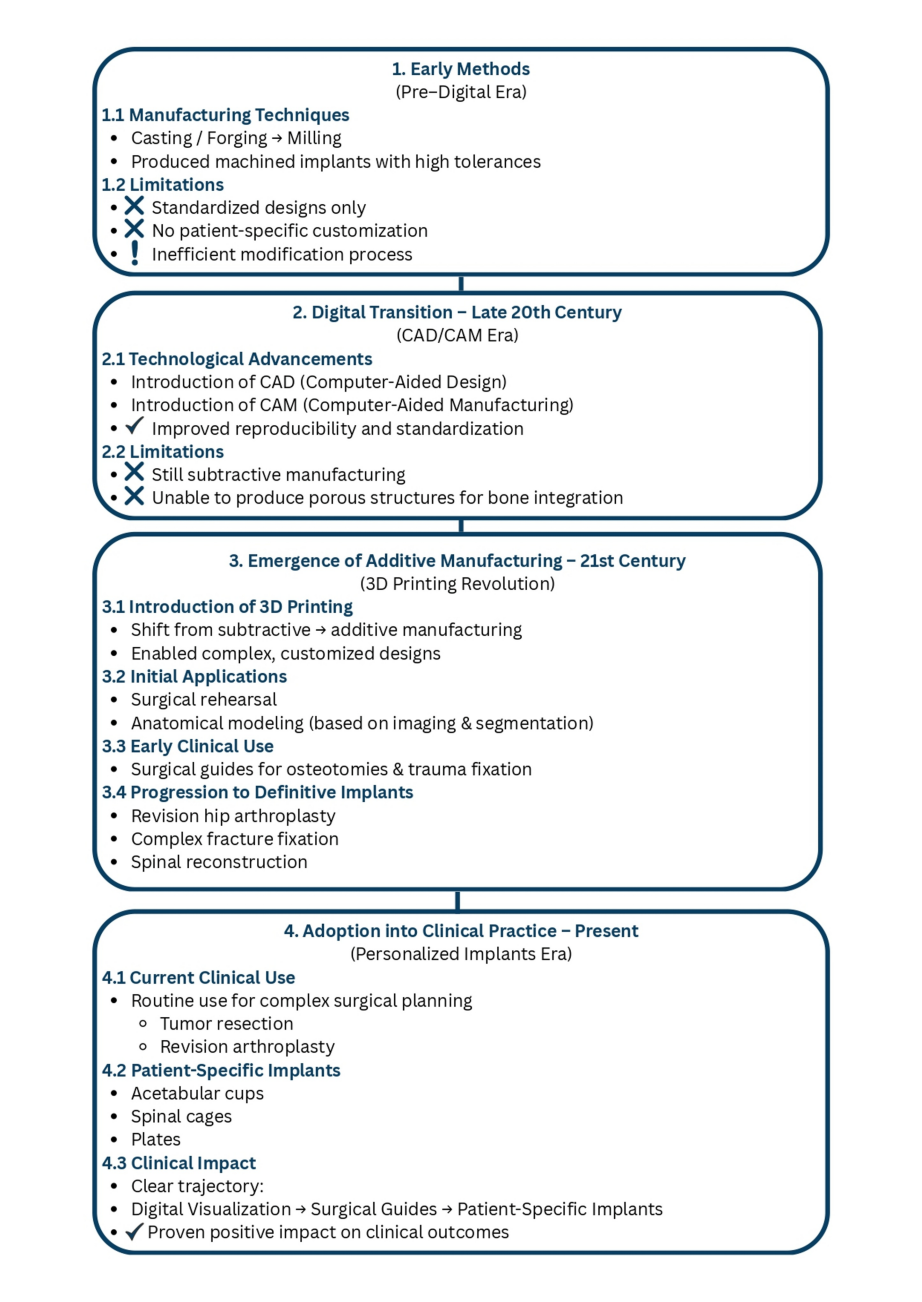
Historical development of implants The figure illustrates the chronological evolution from conventional casting and forging toward CAD/CAM and additive manufacturing. Each transition reflects key technological milestones aimed at improving customization, biocompatibility, and structural integrity. Earlier methods were phased out due to limited adaptability and poor osseointegration potential, whereas 3D printing enabled porous, patient-specific scaffolds to overcome these limitations.

Scoping reviews [9] illustrate how 3D printing technology has evolved from being a rare experimental use in special cases to everyday medical practice in high-volume settings. Complex orthopedic cases (e.g., revision arthroplasty or tumor resection) have been planned on 3D-printed anatomical models in modern times. More and more definitive implants, in particular acetabular cups, spinal cages, and patient-specific plates, are made using additive fabrication. This historical development of 3D printing in orthopedics as a field demonstrates a wider development trajectory - from digital visualization to surgery guides to patient-specific implantations - along with growing evidence indicating its proven influence on clinical outcomes. Figure 1 illustrates the complete history of the evolution of 3D printing in orthopedic practice.

There are various 3D-printing technologies used in orthopedic applications, and the various properties depend on material, precision, and intended use summarized in Table 1.

**Table 1:** Types of 3D printing technologies used in orthopedics The table summarizes the primary additive manufacturing modalities, their mechanisms, and clinical applications. The “Limitations/Considerations” column has been expanded to include reproducibility challenges, material-specific restrictions, and regulatory concerns that influence clinical translation. PLA: Polylactic acid; PCL: Polycaprolactone.

Technology	Method	Applications	Key Properties/Advantages	Limitations/Considerations
Selective laser melting (SLM)	Uses a laser to selectively fuse metallic powders layer by layer.	Manufacturing titanium alloy implants (acetabular cups and spinal cages).	Facilitates mechanical structure-reassociation coupling and osseointegration. High accuracy and mechanical properties.	Metallic powders, layer-by-layer fusion.
Electron beam melting (EBM)	Uses an electron beam to fuse metallic powders. Works at high build temperatures.	Larger orthopedic devices and titanium implants.	Reduces residual stress in titanium implants. High accuracy and mechanical properties.	High build temperatures.
Stereolithography (SLA)	Uses a UV laser to cure photopolymer resins.	Anatomical models and surgical guides (preoperative planning for trauma and tumor surgery).	Very useful for generating anatomical models and surgical guides.	Less commonly used for definitive implants due to material biocompatibility.
Fused deposition modeling (FDM)	Extrusion of thermoplastic filaments (PLA and PCL).	Teaching models, surgical practice, and experimental biodegradable implant studies. Potential for pediatric implants.	Cost-effective. Widely used in teaching and experimental studies.	Not appropriate for load-bearing implants.

Selective Laser Melting (SLM)

SLM uses a laser to selectively fuse metallic powders layer by layer. It is also extensively employed for manufacturing titanium alloy implants, such as acetabular cups and spinal cages. SLM facilitates the mechanical structure-reassociation coupling and osseointegration through this specific process [10].

Electron Beam Melting (EBM)

Similar to SLM, EBM uses an electron beam in place of a laser. Compared to conventional methods, as such may reduce residual stress in titanium implants; it works at high build temperatures, which allows it to be employed especially with larger orthopedic devices. SLM and EBM account for the bulk of the production of definitive metallic implants because of their accuracy and mechanical properties.

Stereolithography (SLA)

SLA uses a UV laser to cure photopolymer resins. SLA is less commonly used for definitive implants because of material biocompatibility, but it is very useful for generating anatomical models and surgical guides. Surgeons frequently use SLA models in preoperative planning for trauma and tumor surgery [11].

Fused Deposition Modeling (FDM)

FDM consists of extrusion of thermoplastic filaments (i.e., polylactic acid (PLA) and polycaprolactone (PCL)). Although not appropriate for load-bearing implants, FDM is cost-effective and has been widely used in teaching models, surgical practice, and experimental biodegradable implant studies. Developments in biodegradable polymer printing could extend FDM to be used in pediatrics, where implants need to be only temporarily placed.

Materials in 3D-printed orthopedic implant selection are as important as the printing technology, as they are directly related to biocompatibility, osseointegration, mechanical strength of the implant, and long-term implant survivorship.

Titanium and Titanium Alloys

Titanium alloys such as Ti6Al4V continue to be the basis of 3D-printed orthopedic implants. The high mechanical properties (high strength-to-weight ratio and corrosion resistance) and high biological compatibility together make them popular. The excellent ability to fabricate implants with highly porous surfaces through SLM or EBM makes titanium well suited for promoting bone ingrowth [12]. This porous scaffold minimizes stress shielding compared to dense machined implants and offers an environment favorable for osteoblast adhesion and vascularization.

Biodegradable Polymers

Biodegradable polymers, especially poly(lactic-co-glycolic acid) (PLGA), are attracting great interest. With the addition of calcium composites, PLGA scaffolds have better osteoconductivity and mechanical properties for bone regeneration in load-sharing applications [12]. These composites degrade over time, minimizing the need for implant removal, especially beneficial in pediatric orthopedics, where growth requires revision surgeries.

New Biodegradable Orthopedic Implants

A more comprehensive type of biodegradable implants, which also includes magnesium alloys, PLA, and PCL, is being studied. Kotteda et al. [13] underlined the potential of such implants to redefine clinical practice by alleviating long-term side effects of permanent implants like stress shielding and abolishing revision surgical procedures. Despite their potential, clinical applications are hindered by issues related to mechanical integrity and degradation control.

Summary

**Figure 2:**
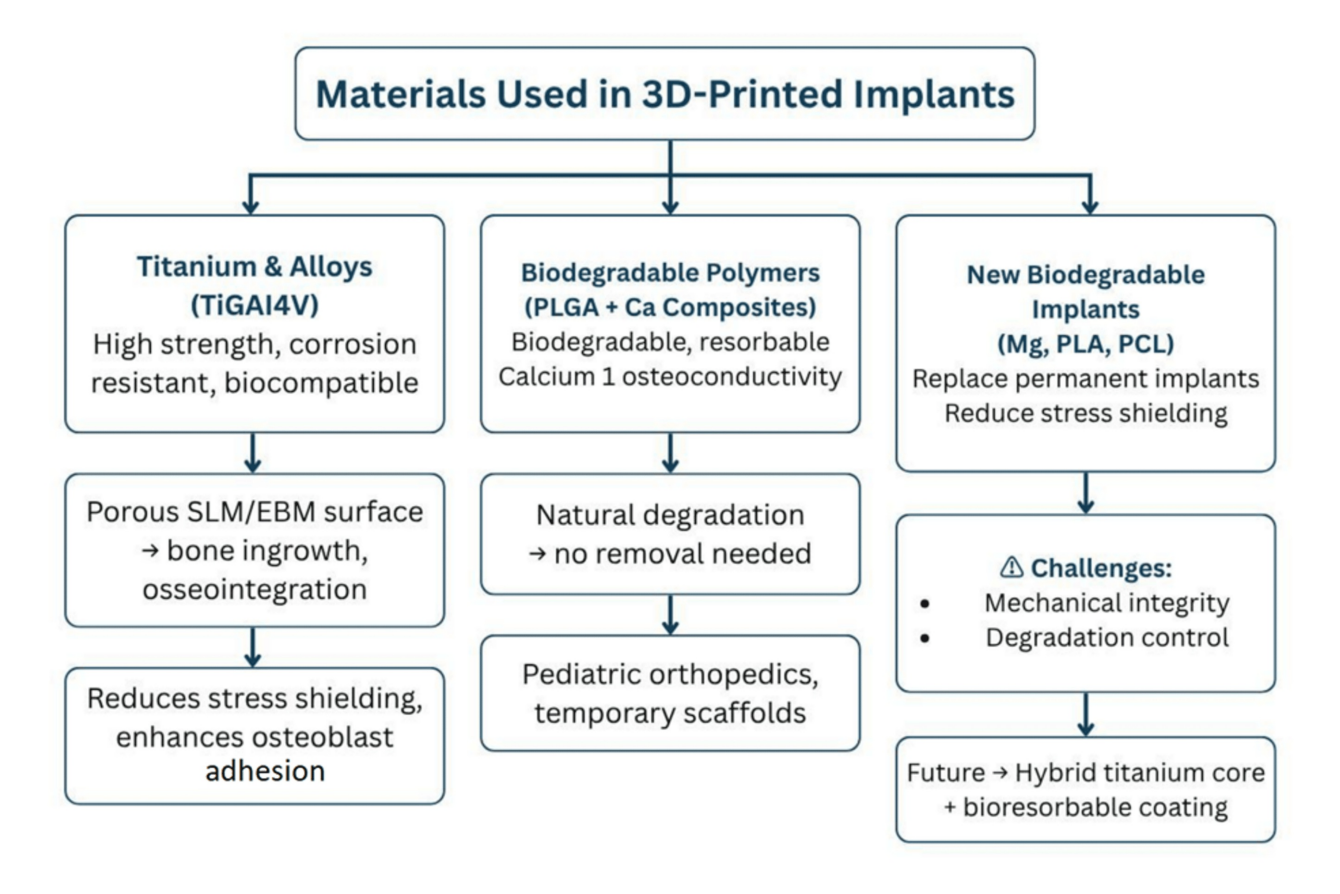
Materials in 3D-printed orthopedic implants PLGA: Poly(lactic-co-glycolic acid); PLA: Polylactic acid; PCL: Polycaprolactone; SLM: Selective laser melting; EBM: Electron beam melting.

Titanium alloys dominate for durable, load-bearing implants, as shown in Figure 2. But biodegradable polymers and composites have received increasing attention for temporary, resorbable scaffolds. The future is likely to lie in hybrid implants that integrate the permanent structural cores of a titanium-based material with bioresorbable coatings or composites to optimize healing.

Comparative outcomes: 3D-printed vs. conventional implants

The clinical implementation of 3D-printed implants has increasingly been compared by cost of devices, durability, complications, and patient satisfaction with conventional orthopedic equipment. Recent systematic evidence shows that 3D-printed implants always result in better anatomic accuracy and operative quality than conventional systems. Roy et al. [14] found that 3D-printed implants increase intraoperative efficiency and reduce surgical time. They are greatly associated with higher patient satisfaction as compared with traditional ones, from making use of a personalized fit, but have the same complication rates as conventional implants. These results are reinforced by clinical assessments of complex orthopedic reconstruction. When comparing 3D-printed implants and implants made with other devices, the mean operative time was reduced, per the studies reviewed, from 15% to 25%, and the alignment accuracy was much better than conventional systems [14,15]. Similarly, patient satisfaction scores on measures of function (Harris Hip Scores or Oswestry Disability Index) on complex reconstructions were always better for the average patient, 10%-20% better [14,16]. Although these results confirm the accuracy and patient comfort benefits of 3D-printed implants, there are few large randomized trials to analyze in the current analysis. O’Brien et al. [15] reported that 3D-printed patient-specific surgical implants had superior function and patient satisfaction, despite greater initial costs compared to standard implants. It is significant to note that long-term economic evaluations revealed a net benefit as 3D-printed devices saved the need for revision surgeries. Likewise, in the oncologic setting, Mounsef et al. [16] carried out a combined meta-analysis showing better functional properties and decreased implant-related complications when comparing 3D-printed reconstruction methods to surgical techniques. Studies in arthroplasty have produced similar results. Barakeh et al. [17] established that 3D-printed hip implants not only lower stress shielding but also lengthen implant life and improve postoperative recovery trajectories. Collectively, these data imply that while conventional implants are economical in simple cases, 3D-printed devices present better results in anatomically complex or revision procedures. And in all these contexts, it could be observed that the initially more expensive process is compensated for by long-term durability, lower rates of complications, and therefore higher patient satisfaction.

Economic Considerations: Cost-Effectiveness of FDM

The printing technology underlying AM has a lot to do with its financial feasibility. From these solutions, FDM has become one of the most cost-effective methods to produce surgical models and patient-specific implants. Czyżewski et al. [18] pointed out that FDM-based cranioplasty products were significantly lower in cost than traditional implants, yet retained similar safety and functional status. They stressed the fact that low-cost thermoplastics employed in FDM substantially reduced production expenses, rendering it a feasible choice in low-resource healthcare systems. Further evidence is found indicating long-term cost savings of FDM. FDM-generated surgical models resulted in reduced operating times and intraoperative complications as demonstrated by Vitali et al. [19]. While the initial investment in 3D-printing infrastructure was substantial, cumulative cost analysis revealed net financial benefits within relatively short time frames. In a wider perspective, Shaikh et al.'s [20] review showed that patient-specific FDM implants reduced material waste, surgical complications, and revision rates when compared to conventional devices. The potential economically sustainable application of FDM in preoperative planning and direct manufacturing, as implemented on-site, is supported by these cost-saving factors, as well as the scalability of the implant economy. The evidence taken as a whole indicates that FDM-based 3D printing could alleviate cost issues due to patient-individualized implants. FDM provides tangible economic benefits, improving access to high-quality care for advanced orthopedic surgery through decreasing per-unit manufacturing costs, shortening operating time, and decreasing revision surgeries.

Applications in orthopedics

3D printing has infiltrated nearly all subspecialties of orthopedics, with unique benefits in advanced reconstructions that cannot be reached with traditional implants.

Hip and Knee Arthroplasty

3D printing is most commonly used in revision hip arthroplasty. Almeida et al. [21] conducted a systematic review and meta-analysis in the area of revision and showed that highly porous titanium acetabular cups obtained by AM could deliver higher biological fixation with less loosening and migration. At the knee level, Zappley et al. [22] described a 10-year follow-up of cementless TKA using 3D-printed materials, corroborating good fixation, adequate alignment, and favorable patient-reported results.

Upper Extremity Applications

In shoulder arthroplasty, patient-specific implants would be valuable, considering that glenoid morphology is highly variable. Wesorick et al. [23] covered recent advances in patient-specific reverse shoulder arthroplasty, with improvements in implant location, decreases in complications, and biomechanical recovery. Similarly, Hua et al. [24] successfully conducted a resurfacing arthroplasty with 3D printing for severely malunited distal humerus with functional recovery and reduction in pain.

Spine Surgery

This has made the spine a center of importance for 3D-printed implants. Ouchida et al. [25] investigated titanium cages for cervical spondylotic myelopathy, finding promising clinical results with good fusion and alignment. For more complex reconstructions, patient-specific vertebral implants can be applied after en bloc spondylectomies successfully [26].

Trauma and Osteotomies

In orthopedic trauma, the accuracy of 3D-printed patient-specific plates and cutting guides has been shown as an important way to increase precision. Oldhoff et al. [27], using 3D-printed drilling guides for corrective osteotomies, showed reduced operative time, improved alignment accuracy, and decreased complication rates.

Oncology

AM has revolutionized orthopedic oncology. Elgamal et al. [28] conducted a systematic review on the effectiveness of 3D printing in sarcoma surgery and highlighted its utility in tumor resection planning and customized reconstructions. Reconstructing large bone defects with specialized implants has enhanced functional and oncological outcomes.

Reported outcomes

The clinical data for 3D-printed orthopedic implants are typically reported in case series, systematic reviews, and small cohort studies and are consistently promising.

Spinal Implants

Aleinikov et al. [26] concluded the biomechanical and clinical outcomes of the patient-specific vertebral implants after en bloc spondylectomy. At two-year follow-up, patients exhibited stable fixation, satisfactory spinal alignment, and higher functional scores, suggesting the feasibility and safety of the intervention as well. Similarly, Ouchida et al. [25] reported good fusion rates with 3D-printed cervical cages.

Complications

Complications still need to be taken into account despite these positive outcomes. Among pelvic sarcoma patients reconstructed with 3D-printed implants, Saberi et al. [29] reported surgical site infections. Although infection rates were not significantly higher compared to conventional implants, the use of these surgical implants faced an important management challenge due to the porosity of the devices.

Systematic Evidence

Roy et al. [14], who have conducted a PRISMA-compliant systematic review, concluded that 3D printing provides an advantage in terms of surgical precision and acute functional results of the implants. Roy et al. [14] confirmed these in a subsequent systematic review and reported both less intraoperative time and hemorrhage. Nevertheless, the shortcoming of these studies was the absence of large and randomized controlled trials that limit current evidence.

General Assessment

Collectively, this evidence supports that 3D-printed implants have performed well, or better, at initial outcomes than conventional implants. They achieved accurate anatomical reconstruction and have been successful in obtaining fast osseointegration, but infection control and long-term durability should be evaluated further.

Limitations and challenges

Despite the good clinical and biomechanical results of 3D-printed implants, with numerous challenges and deficiencies, widespread use of such devices remains a long way off.

Regulatory and Standardization Problems

Standardized manufacturing and regulatory protocols are among the main barriers. This is in contrast to conventional implants that have undergone extremely long, well-established approval processes. Ortis et al. [30] highlighted the lack of standardization of infection control measures in AM workflows (particularly when CAD and reverse engineering are applied). In the absence of international standards, variations in sterilization and porosity control, as well as differences among types of surface finishing, can compromise implant safety and dependability.

Economic Barriers

Cost remains another obstacle to adoption. Malhotra and Johnson [31] found that although 3D printing might bring a cost reduction in very complex reconstructions by reducing operative time and revision surgery, it carries a significant investment in machinery and expertise at the first stage. Also, not all healthcare systems have the necessary infrastructure for in-hospital AM facilities, limiting access to large academic institutions or even tertiary institutions.

Reproducibility and Quality Control

Reproducibility of an experiment is another limitation. Individual differences between machines, machine calibration, powder quality, and build properties may result in very small differences in implant porosity, mechanical strength, and surface features. This variation brings the question of the consistency of manufacturing batches. Although research works [21,22] indicate strong implant survival, reproducibility across various centers is not yet an easy proposition.

Evidence Gap

Last but not least, the shortage of high-level clinical evidence is possibly one of the biggest challenges. The majority of the available trials can be found in case reports, small series, or systematic reviews of early outcomes [14]. Large RCTs and multicenter RCTs are required to prove safety, efficacy, and cost-effectiveness. Until these data are in hand, regulators and investors are going to be skeptical. Another limiting factor is the scalability of AM into healthcare systems. This approach is technically feasible but requires a good deal of investment in infrastructure, quality assurance, and operator training to scale up. The lack of common standards for layer resolution, powder reuse, and post-processing also contributes to variation across production centers. In addition, one of the ethical concerns is that of the security of patient data during CAD modeling, as three-dimensional imaging data constitute sensitive personal information. Regulatory, technical, and ethical gaps have to be filled before full integration into clinical orthopedics.

Future directions

3D printing has the potential to transform the future of orthopedics as it will fall within the intersection of materials science, digital engineering, and biotechnology, with multiple emerging directions.

AI and Digital Adoption

The power of AI is expected to transform 3D-printing design practices. Harale et al. [32] reported how AI can improve implant design by automatically optimizing porosity, geometry, and load distribution according to patient imaging data. Workflows driven by AI could also speed times to manufacture, reduce human failure in segmentation, and customize implants more accurately for a patient.

Shape-Morphing Implants

The concept of shape-morphing implants opens up new avenues beyond traditional, static implants. Moosabeiki et al. [33] proposed additively manufactured implants that respond to anatomical defects in a dynamic manner, focusing mostly on acetabular reconstruction. These solutions may adapt themselves to irregular shapes, minimizing intraoperative manipulation and enhancing long-term fixation.

Innovations in Biologics and Spinal Medicine

The combination of biologics with AM is especially significant for spinal surgery. In this regard, Tian et al. [34] noted the use of biologics together with 3D-printed scaffolds to better enhance osseointegration, decrease pseudoarthrosis, and contribute to better fusion results. Such biologics can, along with 3D-printed structures, become a new frontier in regenerative orthopedics.

Bioprinting and the Engineering of Tissues

And the most exciting frontier, maybe, is bioprinting, which endeavors to make living tissue constructs. Xing et al. [35] provided a review of developments in bioprinting vascularized bone constructs, showing the challenge of generating robust vasculature in 3D-printed scaffolds. Although it would be the ultimate end goal of successful translation of this technology in orthopedic care, replacing invasive implants with personalized, functional bone tissue that was made of living bone that could be used for the patient remains challenging.

Patient-reported outcomes and quality of care

The incorporation of 3D printing technology in orthopedic surgery has been shown to be very effective and promising in the provision of personalized implants and in achieving better surgical accuracy for patients.

Key Findings

Studies repeatedly demonstrate that 3D-printed tailored orthopedic implants lead to significant satisfaction and improve surgical success as well as patient experience [35]. The inherent personalization of 3D printing technology creates a highly personalized fitting device suitable for each individual's body anatomy, which is of pivotal importance in achieving better biomechanical performance along with avoiding associated complications [35].

There are a number of definite advantages mentioned in the literature, such as improved fit and customization, because 3D-printed implants have been designed to deliver a highly custom fit based on the anatomical form of individual patients. This is personalized and contributes to enhancing biomechanical performance and avoiding a high incidence of complications [14]. Additionally, the use of 3D-printed implants, with support of high-tech imaging and careful planning prior to surgery, has been known to drastically reduce both the operative period and the rate of intraoperative errors [14].Moreover, patients who have received 3D-printed implants often report improved satisfaction and quality of life, mainly due to increased stability and enhanced function of the implants. This is frequently followed by shorter recovery times and an observable improvement in the quality of life after surgery [14]. One of the most important beneficial aspects of 3D printing in orthopedics is the ability to create rough-mesh implants and small micropores. It actively assists in the direct implant-endosseous fusion of bone into the patient and osseointegration [36]. Finally, by utilizing individual CT or MRI data, personalized 3D-printed products are designed with the objective of achieving the normal physiological function of human bones to the maximum extent possible while minimizing any negative effects of the implant on the human body [36].

Summary

3D printing has since revolutionized orthopedic surgery, quickly advancing from an experimental method of anatomical modeling to a clinically relevant technology. Evidence supports its application in hip and knee arthroplasty and shoulder reconstruction, spinal cages, trauma fixation, and oncological reconstructions, with several studies documenting increased anatomical fit, enhanced osseointegration, and encouraging short- to mid-term outcomes [21,24,26,28].However, the main limitations remain. Higher costs, varying quality control regulations, and regulatory uncertainty hamper wide-scale implementation [30,31]. In addition, case reports and systematic reviews have been widely used in the existing literature, highlighting that larger randomized controlled studies are required to confirm long-term safety and effectiveness [14]. Thus, on the horizon, the coupling of AI-based design, shape-morphing implants, biologics, and bioprinting holds the potential for the advent of smart, patient-based, and regenerative orthopedic devices [32-35]. If these technologies can reach their full potential, 3D printing could become more than a simple alternative manufacturing process, but will be an integral part of personalized and regenerative orthopedics. As Zimnoch et al. [37] explained about knee arthroplasty, the industry is teetering on the edge of a revolution. The challenge now is to bridge the gap between technical innovation and clinical evidence, leading to technology advances in AM, yielding lasting improvements for patients worldwide.

## Conclusions

Orthopedic surgery has been transformed through 3D printing from a traditional "off-the-shelf" service into a bespoke and precision service. The technology has moved beyond the stage of being used solely as a preoperative planning and anatomy modeling tool to now being placed into clinical use with additively manufactured patient-specific implants. The applications of 3D-printed implants have repeatedly shown good anatomic fitness, short surgical times, and better osseointegration and function recovery versus traditional prosthesis. Applications have since broadened across nearly every orthopedic subspecialty of arthroplasty and spinal surgery, trauma fixation, and oncologic reconstruction, with a substantial number of case series reporting clinical experience. Despite these breakthroughs, challenges persist. Yet, lack of uniform manufacturing standards, expensive upfront investment, and restricted access to in-hospital printing facilities are barriers to clinical applicability. Furthermore, the majority of reported experience is confined to small series and short-to-mid-term follow-up, which shows the need for large-scale, multicenter, randomized clinical trials in order to generate not only long-term safety but also durability and cost-effectiveness. Regulation will, after all, also have to change to ensure confidence and reproducibility for patient safety across the different AM systems.

Though short- to mid-term results are promising, the area is limited by the lack of multicenter, randomized controlled trials available in the field. For the long-term safety of such treatment and value for money, there is a need for global coordinated research. Standardized protocols are also needed for validation of AM processes, quality control, and protection of patient data. Only with this evidence-based expansion will 3D printing be able to move from high-potential innovation to an established standard in the field of orthopedic implantology. In the next few years, orthopedic 3D printing will be a place where digital engineering, biomaterial science, and biotechnology come together. AI integration could further refine and automate implant design; an adaptive device could be the result through shape-morphing implants, which exhibit bioresponsive changes; bioprinting and regenerative medicine will continue to step forward in building living vascularized bone tissue. When applied in practice at the municipal scale, these ideas might inspire an orthopedic surgical revolution in which we move beyond mechanical reconstruction to a biologically integrated approach for regeneration.
